# The molecular mechanisms of regulatory T cell immunosuppression

**DOI:** 10.3389/fimmu.2012.00379

**Published:** 2012-12-14

**Authors:** Kendall A. Smith

**Affiliations:** Division of Immunology, Department of Medicine, Weill Medical College, Cornell UniversityNew York, NY, USA

Fifty years ago Jacques Miller devised a technique to thymectomize neonatal mice to explore the hypothesis that the thymus played a role in the development of immunity. He found that if thymectomized by day 3 postpartum, the mice would develop normally for the first month, but thereafter they underwent a runting syndrome similar to that observed during Graft vs. Host Disease (GvHD) (Miller, 1962). During the second month of life the mice would lose weight and suffer from a dermatitis and generalized lymphadenopathy and splenomegaly followed by premature death. A more detailed examination of the immune system revealed that early after thymectomy the mice were lymphopenic and immunocompromized, unable to reject allogeneic or even xenogeneic skin grafts, and incapable of generating antibodies to routine antigens. Miller correctly interpreted his findings as evidence that the thymus appeared to be critical in the first few weeks of life for the development of a mature functional immune system, but he did not speculate on the cause of the later enigmatic development of lymphoproliferation and apparent autoimmunity.

Twenty years after Miller's seminal observations, Shimon Sakaguchi reported that the lymphoproliferative/autoimmune diseases of immunocompromized day-3 thymectomized (d3Tx) could be transferred to neonatal mice with Thy-1+, Lyt-1+, Ly-23- splenocytes from the afflicted animals (Sakaguchi et al., 1982b). Furthermore, the autoimmune syndrome that developed in d3Tx mice could be completely prevented by a single intraperitoneal injection of Thy-1+, Lyt-1+, Lyt-23- splenocytes or thymocytes taken from normal adult mice (Sakaguchi et al., 1982a). Prior to these experiments, alloantisera reactive with Lyt-1 were thought to mark the helper T cell subset (Th cells) (Cantor and Boyse, 1975; Kisielow et al., 1975). However, Lyt-1 alloantigens were subsequently found on all T cells to a varying degree and therefore could not be the murine equivalent to the T4 (CD4) determinants that specifically identified human Th cells, restricted to antigen recognition with MHC class II molecules (Reinherz et al., 1979b; Ledbetter et al., 1980).

Additional progress in the molecular understanding of the regulation of adaptive immunity was required before it was possible to make further progress in the dissection of these phenomena, especially the molecular mechanism(s) responsible for the apparent suppressive activities of mature T cells vs. neonatal T cells. Thus, T cell clones (Baker et al., 1979) were necessary to define the molecular nature of the T cell antigen receptor (TCR) complex, including the roles of the accessory molecules CD4 and CD8 as facilitating recognition of antigenic peptides bound to MHC class II and class I, respectively (Reinherz et al., 1979a, 1980a), as well as the role of the CD3 molecules as triggers of antigen recognition (Reinherz et al., 1980b), found to be mediated by the disulfide-linked heterodimeric α and β chains (Meuer et al., 1983). Thus, antigen-specific recognition by the TCR complex leads to the expression of antigen-non-specific cytokines, such as interleukin-2 (IL-2) and its receptors (Meuer et al., 1984), so that the tempo, magnitude, and duration of immune responses came to be understood to depend upon antigen non-specific hormone-like molecules (Cantrell and Smith, 1984; Smith, 1988). Inherent in these concepts was the demonstration that IL-2 interacts with specific receptors that satisfied all of the characteristics of true hormone receptors, i.e., high affinity, stereospecificity, saturability, and physiologic relevance (Robb et al., 1981).

Given these findings, a totally unexpected result of the deletion of the IL-2 gene was reported by Ivan Horak's group (Schorle et al., 1991). Mice developing with the total absence of IL-2 were remarkably similar to Miller's neonatal thymectomized mice. Intitially, the IL-2(−/−) mice grew normally and as expected were immunocompromized (Kundig et al., 1993). However, as they aged there occurred a lymphoroliferative syndrome with the accumulation of activated T cells in secondary lymphoid organs and even invasion of non-lymphoid organs that culminated in premature death due to autoimmune hemolytic anemia and inflammatory bowel disease (Horak et al., 1995; Sadlack et al., 1995).

Concurrent with these publications, Sakaguchi and his colleagues reported that a critical subset of CD4+ T cells that express the IL-2R α-chain, ~10% of mature peripheral CD4+ T cells, could prevent autoimmune diseases of immunodeficient *nu/nu* mice injected with immunocompetent CD4+ T cells depleted of IL-2Rα+ cells (Sakaguchi et al., 1995). Subsequently, the inhibitory molecule CTLA-4 was found to play a major role in the regulatory function of CD4+IL-2Rα+ cells (Takahashi et al., 2000).

The finding that CD4+IL-2Rα+CTLA-4+ cells express the transcriptional regulator FOXP3 helped to explain the phenotype of regulatory T cells (T-Regs) (Fontenot et al., 2003; Hori et al., 2003; Walker et al., 2003). Moreover, IL-2 was found to be required for FOXP3 expression and the normal development of FOXP3+ cells (Zorn et al., 2006; Burchill et al., 2007). Also, FOXP3 was found to inhibit IL-2 expression, which accounted for T-Reg anergy, and led to the conclusion that IL-2 activates a negative-feedback loop via FOXP3 that limits T cell proliferative expansion during an immune reaction (Popmihajlov and Smith, 2008). However, the FOXP3-induced increase in the expression of both CTLA-4 and IL-2Rα chains did not immediately translate into mechanisms that could readily explain immunosuppression (Wu et al., 2006).

A seminal breakthrough in understanding the molecular mechanisms of T-Reg immunosuppression was contributed by Pushpa Pandiyan and Michael Leonardo and their co-workers, who detailed how T-Reg cells, incapable of producing IL-2, are very efficient in binding and degrading IL-2, thereby leading to cytokine deprivation apoptosis of T-Effector cells (T-Eff) (Pandiyan et al., 2007), as well as T-Regs themselves (Pandiyan and Lenardo, 2008).

Thomas Hofer's group (Busse et al., 2010) and independently, Gregoire Altan-Bonnet's group (Feinerman et al., 2010), using both theoretical and experimental approaches, reported that during an immune response there is a competition for IL-2 between T-Regs and activated effector T cells (T-Effs). Moreover, Altan-Bonnet showed that the IL-2 up-regulation of the IL-2Rα+ chain, first noted soon after the IL-2Rα+ chain was discovered (Leonard et al., 1982; Smith and Cantrell, 1985), can result in a 1000-fold increase in the affinity of IL-2 binding to the trimeric IL-2R. Consequently, T-Regs can rapidly respond to the initial IL-2 produced by T-Effs, and up-regulate IL-2Rα+ chains, which will favor IL-2 binding and degradation much more efficiently than T-Effs, which require several hours before they can express IL-2Rα+ chains upon antigen stimulation. Thus, the “strength” of the initial antigenic stimulation, which determines the amount of IL-2 produced initially, can be overcome by T-Regs when the antigens are of low affinity or at low concentrations (i.e., “weak”), but cannot be competed successfully by T-Regs if the antigenic stimulus is “strong” (i.e., high affinity or at high concentrations). Assuming autoantigens to be “weak” and non-self antigens to be “strong,” this system could account for self–non-self recognition.

With this brief chronology as background, readers will find many of the contributions to this volume remarkable, in that many of the field leaders, but not all, have reached a consensus that the major molecular mechanism whereby T-Regs suppress T-Effs revolves around their capacity to regulate the availability of IL-2 as well as other cytokines.
